# Complete Genome Sequence of a Common Midwife Toad Virus-Like Ranavirus Associated with Mass Mortalities in Wild Amphibians in the Netherlands

**DOI:** 10.1128/genomeA.01293-14

**Published:** 2014-12-24

**Authors:** Steven J. van Beurden, Joseph Hughes, Bernardo Saucedo, Jolianne Rijks, Marja Kik, Olga L. M. Haenen, Marc Y. Engelsma, Andrea Gröne, M. Helene Verheije, Gavin Wilkie

**Affiliations:** a Faculty of Veterinary Medicine, Utrecht University, the Netherlands; b MRC-University of Glasgow Centre for Virus Research, Glasgow, United Kingdom; c Dutch Wildlife Health Centre, Utrecht, the Netherlands; d Central Veterinary Institute, Wageningen UR, Lelystad, the Netherlands

## Abstract

A ranavirus associated with mass mortalities in wild water frogs (*Pelophylax* spp.) and other amphibians in the Netherlands since 2010 was isolated, and its complete genome sequence was determined. The virus has a genome of 107,772 bp and shows 96.5% sequence identity with the common midwife toad virus from Spain.

## GENOME ANNOUNCEMENT

Several recent amphibian die-offs worldwide have been found to be associated with ranaviruses of the family *Iridoviridae* (1). The ranavirus emerging in Spain since 2007 was tentatively named common midwife toad virus (CMTV) after one of its host species (2). In 2010, a CMTV-like virus was detected during a mass mortality event affecting wild water frogs (*Pelophylax* spp.) in Dwingelderveld National Park, the Netherlands (3). We tentatively designated the virus CMTV-NL and analyzed the temporal spatial and disease patterns in various amphibian hosts (Rijks et al., unpublished data). In order to understand the relationship of CMTV-NL with other ranaviruses, we determined and analyzed its complete genome sequence.

CMTV-NL was isolated from a typically affected fresh dead wild edible frog (*Pelophylax* kl. *esculentus*) collected during an outbreak in a small semiartificial pond in Westerveld, the Netherlands, in August 2013. The affected frogs showed hemorrhagic disease with hepatomegaly and splenomegaly. Microscopically, the liver and kidneys presented cells with basophilic intracytoplasmic inclusion bodies. A 10% (wt/vol) suspension was prepared from a pool of internal organs (heart, liver, spleen, kidney, and tongue) and inoculated on Bluegill fry (BF-2) cells in a CO_2_ incubator at 22°C. Upon the appearance of cytopathic effect, the supernatant was cleared by centrifugation and loaded onto a 36% sucrose cushion in phosphate-buffered saline (PBS) (wt/vol) and centrifuged for 1 h at 20,000 × g at 4°C, as described previously (4). The virus pellet was resuspended in PBS, and DNA was extracted using the DNeasy kit (Qiagen). DNA was sheared by sonication and processed for sequencing by using a KAPA library preparation kit (KAPA Biosystems). A MiSeq platform running v3 chemistry (Illumina) was used to generate 13,127,123 paired-end 300-bp reads. *De novo* assembly of a subset of the data using ABySS (5) resulted in the complete genome sequence, with an average coverage of 8,290 reads per nucleotide. iCORN2 (6) was used to improve the draft genome by correcting errors in the consensus sequence. PCR amplification and Sanger sequencing were performed to confirm repetitive regions in the assembly. RATT (7) was used for generating annotations using the complete genome sequence of the Spanish CMTV isolate *Mesotriton alpestris*/2008/E (CMTV-E) as a reference (GenBank accession no. JQ231222.1) (4).

The genome of CMTV isolate *Pelophylax* kl. *esculentus*/2013/NL is 107,772 bp in length and has a G+C content of 55.3%. Transferring the genome annotation of CMTV-E resulted in the identification of all 104 putative open reading frames for CMTV-NL, although some open reading frames appeared to be truncated. Putative protein functions were predicted based on sequence homology using BLASTp searches (data not shown). The genome of CMTV-NL is fully colinear with those of CMTV-E and *Andrias davidianus* ranavirus isolate 1201 (ADRV-1201) (GenBank accession no. KC865735.1) (8) and demonstrates overall sequence identities of 96.5 and 94.1%, respectively. Phylogenetic analysis of the 26 iridovirus core proteins of available complete ranavirus genomes placed CMTV-NL in a group together with CMTV-E and ADRV-1201, and an analysis of the nucleotide sequences of the major capsid protein placed *Rana esculenta* virus, Zuerich *Pelophylax* collection ranavirus, and pike-perch iridovirus in the same group (data not shown).

### Nucleotide sequence accession number.

The complete genome sequence of CMTV isolate *Pelophylax* kl. *esculentus*/2013/NL has been deposited in GenBank under the accession no. KP056312.
